# Barriers to hydroxyurea use from the perspectives of providers, individuals with sickle cell disease, and families: Report from a U.S. regional collaborative

**DOI:** 10.3389/fgene.2022.921432

**Published:** 2022-08-26

**Authors:** Marsha J. Treadwell, Lisa Du, Neha Bhasin, Anne M. Marsh, Theodore Wun, M. A. Bender, Trisha E. Wong, Nicole Crook, Jong H. Chung, Shannon Norman, Nicolas Camilo, Judith Cavazos, Diane Nugent

**Affiliations:** ^1^ Division of Hematology, Department of Pediatrics, University of California, San Francisco, San Francisco, CA, United States; ^2^ UCSF Benioff Children’s Hospital Oakland, Oakland, CA, United States; ^3^ Division of Hematology/Oncology, Department of Pediatrics, University of Wisconsin School of Medicine and Public Health, Madison, WI, United States; ^4^ Division of Hematology and Oncology, Department of Internal Medicine, University of California, Davis, Davis, CA, United States; ^5^ Odessa Brown Children’s Clinic, Seattle Children’s Hospital, Seattle, WA, United States; ^6^ Division of Pediatric Hematology and Oncology and Department of Pathology, Oregon Health and Sciences University, Portland, OR, United States; ^7^ Center for Inherited Blood Disorders, Orange, CA, United States; ^8^ Hematology-Oncology, Department of Pediatrics, University of California, Davis, Davis, CA, United States; ^9^ Alaska Bleeding Disorders Clinic, Anchorage, AK, United States; ^10^ St. Luke’s Children’s Cancer Institute, Boise, ID, United States

**Keywords:** sickle cell disease, barriers to adherence, disease modifying therapies, models -adherence, hydroxyurea

## Abstract

Sickle cell disease (SCD) is an inherited blood disorder that affects about 100,000 people in the U.S., primarily Blacks/African-Americans. A multitude of complications negatively impacts quality of life. Hydroxyurea has been FDA approved since 1998 as a disease-modifying therapy for SCD, but is underutilized. Negative and uninformed perceptions of hydroxyurea and barriers to its use hinder adherence and promotion of the medication. As the largest real-world study to date that assessed hydroxyurea use for children and adults with SCD, we gathered and analyzed perspectives of providers, individuals with SCD, and families. Participants provided information about socio-demographics, hospital and emergency admissions for pain, number of severe pain episodes interfering with daily activities, medication adherence, and barriers to hydroxyurea. Providers reported on indications for hydroxyurea, reasons not prescribed, and current laboratory values. We found that hydroxyurea use was reported in over half of eligible patients from this large geographic region in the U.S., representing a range of sickle cell specialty clinical settings and practices. Provider and patient/caregiver reports about hydroxyurea use were consistent with one another; adults 26 years and older were least likely to be on hydroxyurea; and the likelihood of being on hydroxyurea decreased with one or more barriers. Using the intentional and unintentional medication nonadherence framework, we found that, even for patients on hydroxyurea, challenges to taking the medicine at the right time and forgetting were crucial unintentional barriers to adherence. Intentional barriers such as worry about side effects and “tried and it did not work” were important barriers for young adults and adults. For providers, diagnoses other than HgbSS or HgbS-β0 thalassemia were associated with lower odds of prescribing, consistent with evidence-based guidelines. Our results support strengthening provider understanding and confidence in implementing existing SCD guidelines, and the importance of shared decision making. Our findings can assist providers in understanding choices and decisions of families; guide individualized clinical discussions regarding hydroxyurea therapy; and help with developing tailored interventions to address barriers. Addressing barriers to hydroxyurea use can inform strategies to minimize similar barriers in the use of emerging and combination therapies for SCD.

## Introduction

Sickle cell disease (SCD) is an inherited blood disorder that affects the hemoglobin of red blood cells (Piel et al., 2017). Sickle cell genotypes include HgbSS, HgbSβ^0^ or Sβ^+^thalassemia, and HgbSC, with varying levels of disease severity (Ballas et al., 2012). In the U.S., about 100,000 people have SCD, with about 1 in every 365 African-American infants born with the disease (Centers for Disease Control and Prenvention, 2020). The sickling of red blood cells secondary to HgbS polymerization leads to a variety of pathophysiological consequences such as inflammation, oxidative stress, hemolytic anemia, hypoxia, and hypercoagulability (Chakravorty and Williams, 2015; Piel et al., 2017) which can result in vaso-occlusion and ischemia. Clinically, SCD can cause severe pain associated with vaso-occlusive episodes (VOE), splenic sequestration, acute chest syndrome (ACS), bacterial sepsis, ischemic strokes and silent cerebral infarcts (Chakravorty and Williams, 2015; Piel et al., 2017).

Recurrent and repetitive VOEs may lead to frequent healthcare visits or interference with daily functioning, even in the absence of healthcare utilization, that negatively affect the quality of life for children and adults with SCD [National Heart, Lung and Blood Institute, National Institutes of Health (NHLBI, NIH), 2014; Jerrell et al., 2011; Tanabe et al., 2012]. Long-term management of SCD requires chronic medical care with access to education about prevention as well as current and future disease modifying and curative therapies (Wang et al., 2011; Alvarez et al., 2013; Yates et al., 2013; DeBaun et al., 2014; Lebensburger et al., 2015; McGann and Ware, 2015; Fitzhugh et al., 2017; Niihara et al., 2018; Quinn, 2018; Krishnamurti et al., 2019). In addition to hydroxyurea, other recently FDA approved therapies include voxelotor, L-glutamine, and crizanlizumab (Ataga et al., 2017; Howard et al., 2019; Vichinsky et al., 2019). Other disease modifying therapies in clinical trials include fetal hemoglobin inducers (Molokie et al., 2017), anti-inflammatory agents (Field et al., 2017a, 2017b; Hoppe et al., 2017; Daak et al., 2018), anti-platelet agents (Hsu et al., 2018), pyruvate kinase activators, heme binders, and anti-adhesion agents (Ataga et al., 2017).

Although the recently approved and investigational therapies hold the promise of better outcomes for people living with SCD, hydroxyurea has been FDA approved since 1998 as a disease-modifying therapy for SCD (Ault, 1998) but is underutilized. The U.S. National Heart, Lung and Blood Institute (NHLBI) guidelines recommend that all individuals with HgbSS or HgbSβ^0^ thalassemia be offered hydroxyurea, beginning at the age of 9 months (National Heart, Lung and Blood Institute, National Institutes of Health, 2014). Further, eligibility criteria for hydroxyurea use in adults and children include: the experience of three or more sickle cell-related moderate to severe pain episodes in a 12-month period; pain that interferes with daily activities and quality of life; history of severe and/or recurrent acute chest syndrome (ACS); or severe anemia regardless of the genotype (National Heart, Lung and Blood Institute, National Institutes of Health, 2014). Multiple studies worldwide have demonstrated the benefits of hydroxyurea for the management of SCD (Wang et al., 2011; Lobo et al., 2013; Luchtman-Jones et al., 2016; Rigano et al., 2018; Tshilolo et al., 2019), including its safety for long-term use (Steinberg et al., 2010). However, perceptions of hydroxyurea and barriers continue to hinder full adherence and promotion of the medication for individuals with SCD. These barriers include: providers’ lack of knowledge about hydroxyurea and its indications, manufacturers’ lack of liquid formulations being widely available for children, refill procedures for different pharmacies, and medication costs. Patient/family barriers include: aversion to taking medications, lack of education about hydroxyurea, doubt about its effectiveness, forgetfulness, cost, getting refills on time, and concerns about potential and experienced side effects (Haywood et al., 2011; Badawy et al., 2017; Sinha et al., 2018; Hodges et al., 2020). Because of its origin as a chemotherapy agent (Kennedy, 1972), some have concerns that hydroxyurea use can lead to increased risk of cancers and impact fertility and the fetus of a pregnant woman (Björkholm et al., 2011; Algiraigri and Radwi, 2014; Castro et al., 2014).

Understanding barriers to hydroxyurea use, given its role as a cornerstone for disease modification, will be helpful as newer therapies begin to be utilized. Previous studies have often used small samples or limited age ranges, or were conducted at a single site (Haywood et al., 2011; Badawy et al., 2017; Sinha et al., 2018). A notable exception is a recent study from the NHLBI Sickle Cell Disease Implementation Consortium (SCDIC) that used qualitative data to examine intentional and unintentional hydroxyurea nonadherence as a framework to understand hydroxyurea use (Hodges et al., 2020). Participants (*n* = 90 adults) came from five sites across the U.S. and completed semi-structured interviews about barriers to hydroxyurea adherence. For over half of the participants (*n* = 52, 57.8%) who were currently taking hydroxyurea, nonadherence was most commonly unintentional (70%, i.e., forgetting, competing life demands) versus intentional (30%, i.e., concerns about adverse effects, aversion to taking medications in general). For those who had never taken hydroxyurea (*n* = 12, 13%), participants reported that they were never offered the medication by their providers.

The goal of this study was to describe factors associated with hydroxyurea use from the perspectives of providers, individuals with SCD, and families. Gaps intended to be filled by this analysis include: 1) improving understanding of barriers to hydroxyurea in order to ensure optimal use of newer disease modifying therapies as they come available and may be used alone or in combination with hydroxyurea; 2) utilization of a framework to understand barriers to adherence, to potentially improve educational efforts and tailor interventions to promote adherence; 3) complement data from clinical trials with hydroxyurea with real-world evidence. We describe clinical characteristics, healthcare utilization, barriers to hydroxyurea use and adherence from the perspectives of 412 pediatric and adult patients with SCD. Providers reported on their prescribing patterns and laboratory values in relation to hydroxyurea for eligible patients. Our aims were to first, delineate factors contributing to hydroxyurea utilization for a larger sample size and broader age range in a single study than previously undertaken. Second, our aim was to gain in-depth insights about barriers to hydroxyurea within the framework of intentional and unintentional nonadherence. We established a shared strategy for data capture in routine clinical settings to allow for the assessment of factors associated with hydroxyurea use within a range of clinical practices and settings situated in the diverse geography of seven western states in the U.S.

## Materials and methods

### Participant recruitment and setting

Parents/caregivers (referred to as caregivers hereafter) of children with SCD younger than 18 years and adults with SCD 18 years and older were recruited from nine clinical sites across seven western U.S. states comprising the Pacific Sickle Cell Regional Collaborative (PSCRC, pacificscd.org). The PSCRC is funded by the U.S. Health Resources and Services Administration (HRSA) and uses a regional model to improve access to care for individuals with SCD. At the time of data collection in 2015–2017, the PSCRC sites included three in California—University of California San Francisco (UCSF) Benioff Children’s Hospital Oakland (BCH-Oakland), University of California Davis, and the Center for Inherited Blood Disorders (coordinating center). The six other PSCRC sites were Providence Hospital in Anchorage, Alaska; the University of Arizona Cancer Center in Tucson; St. Luke’s Health System in Boise, Idaho; Children’s Specialty Center Nevada in Las Vegas; Oregon Health and Sciences University in Portland; and Seattle Children’s Hospital/Odessa Brown Children’s Clinic in Washington.

Eligible patients were either mailed a brochure describing the “PSCRC Minimum Dataset” or their healthcare providers described the project at a regular clinic visit. Individuals were eligible for the study if they 1) had a confirmed SCD diagnosis, 2) were followed at one of the nine PSCRC sites, and 3) were eligible for hydroxyurea therapy according to the NHLBI guidelines (National Heart, Lung and Blood Institute, National Institutes of Health, 2014).

### Ethical considerations

Approvals were obtained from BCH-Oakland institutional review board (IRB) and from Western IRB (now WCG IRB, https://www.wcgirb.com) prior to study startup. The remaining clinical sites relied on Western IRB. Once introduced to the study, participants reviewed the study and procedures with trained study staff in a private area of the clinic. Participants were allowed to ask questions and had the opportunity to discuss the study with family or others before providing their written informed consent.

### Study design and data collection

A convenience sample of caregivers of children with SCD and adults with SCD completed a 15–20-min survey with 17 questions at study enrollment for this cross-sectional descriptive study. Data were collected by research staff using an iPad on the hospital network, a clinic desktop, or a paper-based form, and were entered into a secure REDCap database housed at UCSF.

Questions came primarily from the consensus measure of Phenotypes and eXposures (PhenX) Toolkit - Sickle Cell Disease Core (Eckman et al., 2017), supplemented with additional data elements. Participants provided information about socio-demographics, including age, gender, race/ethnicity, annual household income, household density, head of household education, and health insurance. Participants indicated SCD diagnosis, the number of hospital admissions and emergency department (ED) visits for pain (0, 1, 2, 3, or 4 or more) and number of severe pain episodes that interfered significantly with daily activities (less than 4, or 4 or more) in the previous 12 months. For patients prescribed hydroxyurea, caregivers or adults reported current dose and number of days adherent in the past 2 days (0, 1, or 2) (Stirratt et al., 2015). Both those “on” and “not on” hydroxyurea completed a checklist of 12 potential barriers to hydroxyurea use (i.e., forgetting, worry about side effects, heard “scary” things, frequency of monitoring). Providers reported on current dose of hydroxyurea, indications for its prescription, and current laboratory values (i.e., white blood count, total hemoglobin, and hemoglobin F%). Providers specified indications for hydroxyurea prescription from a checklist (recurrent pain, ACS, neurologic, empiric, or other) or for those not on hydroxyurea, the reasons not prescribed (i.e., not indicated, patient/family preference, had to be discontinued).

### Statistical analysis

Descriptive statistics (frequencies, percent, means, standard deviations) were used to characterize demographics, clinical characteristics and patient/caregiver reports of barriers to hydroxyurea, pain interference and healthcare utilization, as well as provider reports of laboratory values, indications for hydroxyurea or reasons not prescribed. Categorical variables were analyzed using chi-square, or Fisher’s exact test for sparse tables. Continuous variables were compared using *t*-test or Mann-Whitney *U* test, as appropriate. Barriers to hydroxyurea were further categorized as those outside of the individual’s/family’s control that might lend to “unintentional nonadherence” (i.e., doctor does not recommend; too many other priorities; don’t know enough about hydroxyurea; challenges with taking medicines on time; forgetting) and barriers that might contribute to “intentional nonadherence” [i.e., not interested in hydroxyurea; worry about side effects; dislike of additional blood tests and/or clinic visits; prefer not to think about SCD when feeling well; “I tried it and it did not work;” and “heard scary things about (hydroxyurea)”] (Hodges et al., 2020).

Univariate analysis was used to evaluate potentially significant variables (*p* = 0.10) that were independently associated with the dependent variables, for inclusion in multivariable models. Separate multivariable logistic regression models were run for the variables of provider reported prescription of hydroxyurea (yes/no) and patient/caregiver report of “currently on hydroxyurea” (yes/no). Variable selection for the final models used stepwise variable selection, with age and gender identity kept in the models as potential confounders. Significance levels were set at *p* = 0.01, to account for multiple testing. If more than 25% of data for a particular measure (i.e., lab values) were missing, the participant was dropped from the analysis. All data analyses were conducted in SAS Version 9.4 (SAS Institute Inc., Cary, NC, United States).

## Results

A total of 412 participants with SCD were enrolled, with a median age of 15 (range 0.75–69 years). Participants were categorized as children 12 years and younger (the predominant age group at 43.2%); adolescents 13–17 years; young adults 18–25 years; and adults 26 years and older (Table 1). Over half (51.5%) identified as female and the majority (90%) identified as Black/African American race, not Hispanic/LatinX ethnicity (91.5%). Almost 44% of participants reported an annual household income of less than $30,000 with an average household density of 3.8 ± 1.9 people per household. The most common educational attainment of the head of household was high school diploma and higher (61.6%). Similar to other SCD populations, 68.2% of patients were publicly insured. The majority were followed at the three California sites (68.7%), while the remainder were followed at the other PSCRC sites in six states.

**TABLE 1:** Participant socio-demographics (*N* = 412)^a^.

Category	n (%)
Age	
Children (≤12 years)	178 (43.2)
Mean ± SD	6.7 ± 3.4
Adolescents (13–17 years)	66 (16.0)
Mean ± SD	15.0 ± 1.4
Young Adults (18–25 years)	54 (13.1)
Mean ± SD	21.1 ± 2.4
Adults (≥26 years)	114 (27.7)
Mean ± SD	38.7 ± 10.6
Gender Identity	
Female	212 (51.5)
Male	200 (48.5)
Race/Ethnicity	
Black/African American race	385 (93.4)
Other race	27 (6.8)
Hispanic/LatinX ethnicity	35 (8.5)
Annual household income	
<$30,000	181 (43.9)
$30,000–$59,999	63 (15.3)
≥$60,000	61 (14.8)
Unknown	107 (25.9)
Highest education completed by head of household	
<High school graduate	141 (34.2)
≥High school graduate	254 (61.6)
Unknown	17 (4.1)
Health insurance^b^	
Public	281 (68.2)
Private	111 (26.9)
Other government-sponsored	94 (22.8)
Other/Unknown	9 (2.2)
State^c^	
California (*n* = 3 sites)	283 (68.6)
Other PSCRC sites (*n* = 6 sites)	129 (31.3)

a Responses were reported by adults with SCD, or caregivers of children with SCD., Some responses (i.e., race/ethnicity and health insurance) are >100% due to multiple responses by adults with SCD, or caregivers of children with SCD.

b Public health insurance includes Medicare and Medicaid/Medi-Cal. Other Government-sponsored health insurance includes State Children’s Health Insurance Program (SCHIP), Military Health Care (Tricare/VA/CHAMP-VA), and state-sponsored health plan. Other health insurance includes Indian Health Service.

c Sites in California included University of California San Francisco Benioff Children’s Hospital Oakland; University of California Davis, and Center for Inherited Blood Disorders. Sites in other states included Children’s Specialty Center of Nevada; University of Arizona Cancer Center (Tucson); Oregon Health and Sciences University; Seattle Children’s Hospital/Odessa Brown Children’s Clinic (Washington); St. Luke’s Health System (Idaho); and Providence Hospital Anchorage, Alaska.

### Patient/caregiver reports: Clinical characteristics, healthcare utilization, barriers to hydroxyurea and adherence

The majority (80.3%) of patients were diagnosed with sickle cell anemia (SCD genotypes SS or HgbSβ^0^ thalassemia) per patient/caregiver report (Table 2). While 41.5% reported no hospital admissions in the previous 12 months, an equal percentage (41.5%) reported 4 or more admissions in the previous 12 months. Almost half of the sample (*n* = 48.8%) reported 4 or more ED visits in the past 12 months and the majority (60.2%) reported fewer than 4 visits for pain managed at home but that interfered with daily functioning in the previous 12 months. Adults (26 years and older) had the highest proportion of 4 or more ED visits for pain (37.5%, *p* = 0.001) and pain episodes interfering at home (57% reporting 4 or more such episodes in the past year (*p* < .0001)) while adolescents (13–17 years) had the highest proportion of 4 or more hospitalizations for pain in the past year (28.8%, *p* = 0.003).

**TABLE 2:** Clinical characteristics, barriers to care and health behaviors (*N* = 412)^a^.

Characteristic	n (%)
Sickle cell disease diagnosis	
HgbSS or HgbSβ^0^ Thal	331 (80.3)
HgbSC	57 (13.8)
Other	24 (5.8)
Hospital admissions for pain, previous 12 months	
0	171 (41.5)
1 - 3	67 (16.3)
4 or more	171 (41.5)
Emergency Department visits for pain, previous 12 months	
0	152 (36.9)
1 - 3	58 ((13.6)
4 or more	201 (48.8)
Severe pain episodes, interfering with daily activities, previous 12 months	
Less than 4	248 (60.2)
4 or more	158 (38.3)
Hydroxyurea use	
Yes	232 (56.3)
No	162 (39.3)
Hydroxyurea adherence	
Not adherent (0/2 days)	23 (9.9)
Partially adherent (1/2 days)	15 (6.4)
Adherent (2/2 days)	187 (80.6)
Barriers to Hydroxyurea Contributing to Unintentional Nonadherence^b^	
No barriers	257 (62.4)
1 barrier	139 (33.7)
2 or more barriers	16 (3.9)
Barriers to Hydroxyurea contributing to Intentional Nonadherence^c^	
No barriers	302 (73.3)
1 barrier	77 (18.7)
2 or more barriers	33 (8.0)
Total Barriers to Hydroxyurea	
No barriers	157 (38.1)
1 barrier	179 (43.4)
2 or more barriers	76 (18.4)

a From patient/caregiver reports.

b Barriers to Care contributing to Unintentional Nonadherence included: doctor does not recommend it; competing priorities; don’t know enough about hydroxyurea; hard to take the medicine at the right time; forgetting.

c Barriers to Care contributing to Intentional Nonadherence included: not interested in another medicine; worry about side effects; don’t like frequent blood tests and/or clinic visits; don’t like to think about sickle cell disease when feeling well; tried and did not work; heard scary things about hydroxyurea.

**FIGURE 1:**
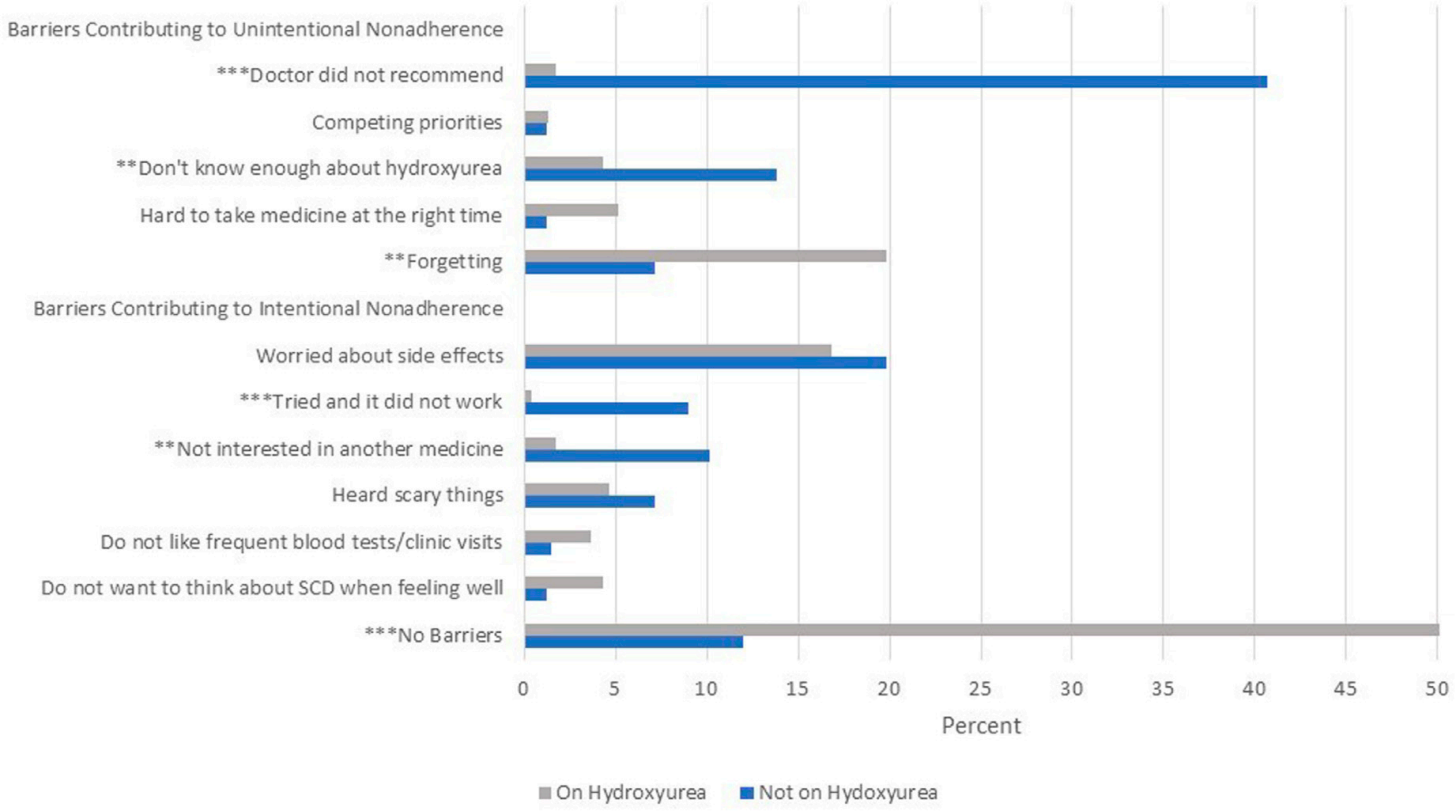
Barriers to hydroxyurea use as reported by adults and caregivers of children with sickle cell disease (SCD). Notes: Side effects worried about included cancer, hair loss, nail discoloration, nausea/dizziness. “Scary” things heard about included cancer, infertility, risk of infection and hair loss. **p < 0.001 ***p < 0.0001.

Out of the 412 eligible participants, 232 participants (56.3%) reported being on hydroxyurea therapy, including 65.2% of children, 62.1% of adolescents, 55.6% of young adults, and 39.5% of adults 26 years and older (*p* = 0.0003). Out of the 232 participants reporting to be on hydroxyurea, 187 (80.6%) reported being completely adherent with taking hydroxyurea over the previous 2 days, while 38 participants (16.3%) reported partial or non-adherence. Twenty eight percent of young adults and adults reported two or more barriers to hydroxyurea, compared with 11.6% of caregivers of children (*p* < 0.0001). Forty-one percent of participants who were not on hydroxyurea reported that their doctors did not recommend it, compared with 1.7% of those on hydroxyurea (*p* < 0.0001, Figure 1). Not knowing enough about the medicine (13.8 vs. 4.3%, *p* < 0.000 = 1), not being interested in taking another medicine (10.2 vs. 1.7%, *p* < 0.001) and “tried it and it did not work” (9 vs. .4%, *p* < 0.0001) were more common barriers for those not on hydroxyurea compared with those who were on it. Forgetting was a more common barrier for those on hydroxyurea (19.8 vs. 7.2%, *p* < 0.001). A common barrier for both participants on and not on hydroxyurea was worry about side effects (16.8 and 19.8% respectively). Over half (52.6%) of participants on hydroxyurea reported no barriers to its use compared with 12% not on hydroxyurea (*p* < 0.0001). The presence of one or more barriers approached significance as negatively associated with complete adherence (*p* = 0.012). There were not significant age differences in reports of barriers contributing to unintentional nonadherence, but young adults and adults were more likely to report barriers that contributed to intentional nonadherence compared with caregivers of children with SCD (38.7% versus 18.4%, *p* = 0.0007).

### Provider reports: Reasons for prescribing or not prescribing hydroxyurea, laboratory values

The number of participants that providers reported were on hydroxyurea (*n* = 236 participants) were consistent with patient/caregiver reports. The most frequent indications reported by providers for prescribing hydroxyurea (Table 3) were recurrent episodes of pain (66.5%) and history of acute chest syndrome (19.9%). Hydroxyurea was prescribed less often for other complications (11.9%) and for neurological complications (7.2%), while it was prescribed for empiric use 9.3% of the time. Of 22 participants who were prescribed hydroxyurea for empiric use, only 1 participant (4.5%) reported that they had experienced 4 or more hospitalizations for pain in the past 12 months. Fewer patients who were prescribed hydroxyurea for empiric use reported 4 or more episodes of severe pain interfering with daily activities in the previous 12 months compared to participants who were not prescribed hydroxyurea for empiric use (*p* = 0.004).

**TABLE 3:** Provider-reported indications & reasons for hydroxyurea prescription^a^.

Category	n (%)
Indications for hydroxyurea prescription (*n* = 236)	
Recurrent Pain Episodes	157 (66.5)
Acute chest syndrome	47 (19.9)
Empiric use	22 (9.3)
Neurological complications	17 (7.2)
Other indication (e.g., anemia, dactylitis)	28 (11.9)
Reasons not on hydroxyurea (*n* = 176)	
No indications	61 (34.7)
Hydroxyurea offered but not interested	40 (22.7)
Hydroxyurea discontinued	29 (16.5)
Chronic transfusion	25 (14.2)
Hydroxyurea not yet been introduced	18 (10.2)
Concerns about adherence to medication/monitoring protocol	4 (2.3)
Other reason (e.g., hemoglobin levels stable, hgbsc or hgbsβ+ genotype, didn’t feel well on hydroxyurea, kidney issues, liver issues, pregnancy)	20 (11.4)
Reasons hydroxyurea discontinued (*n* = 29)	
Patient/family preference	10 (34.5)
Chronic transfusion	9 (31.1)
Side effects	7 (24.1)
Other reason (e.g., not helping patient, pregnancy)	7 (24.1)

a Some responses add up to >100% due to multiple responses.

For the 176 participants not on hydroxyurea, providers reported the medication was not indicated for 34.7% (Table 3), although 38 of the 61 patients had diagnoses of HgbSS or HgbSβ^0^ thalassemia. Reasons for discontinuing hydroxyurea included patient/family preference (34.5%), chronic transfusion therapy (31.1%), side effects (24.1%), or other reasons (24.1%). Providers reported that 22.7% of patients/families offered hydroxyurea were not interested, it had to be discontinued (16.5%) or hydroxyurea had not yet been introduced (10.2%). Providers were concerned about adherence for 7 patients (4.0%).

Providers recorded laboratory data for the study, for 206 participants on hydroxyurea and 106 participants not on hydroxyurea. The total white blood cell count (WBC) and absolute neutrophil count (ANC) were lower (*p* = 0.01 and *p* = 0.002 respectively) and HbF was higher (*p* < 0.001) for participants on hydroxyurea compared to those not on hydroxyurea (Table 4).

**TABLE 4:** Provider-reported laboratory values by hydroxyurea use (*n* = 312)^a^.

Category	On hydroxyurea (*n* = 206) Mean ± SD	Not on hydroxyurea (*n* = 106) Mean ± SD	References range
CBC			
White blood cell count (x10^3^ cells/ul)	10.3 ± 0.3	11.9 ± 0.5	5.0–10.0*
Hemoglobin (g/dL)	9.0 ± 0.1	9.3 ± 0.2	13.5–17.3 (M)
			12.0–15.5 (F)
Platelet count (x10^3^ cells/ul)	349.8 ± 9.2	352.0 ± 14.8	150.0–400.0
Absolute neutrophil count (x10^3^/ul)	5.1 ± 0.2	6.5 ± 0.4	2.0–8.0*
Hb f (%)	15.6 ± 0.9	7.9 ± 1.1	<2.5%**
Liver			
Bilirubin, total (mg/dL)	2.5 ± 0.1	2.7 ± 0.2	0.0–1.2
ALT (U/L)	28.0 ± 1.6	31.4 ± 3.0	0.0–41.0 (M)
			0.0–33.0 (F)

*p < 0.01, **p < 0.001.

### Multivariable models for hydroxyurea use/prescription

Based on results from univariate models, diagnosis, HbF, indications for hydroxyurea prescription (empiric use, recurrent pain), total barriers and barriers contributing to unintentional nonadherence were entered into a multivariable model for patient/caregiver reports of being on hydroxyurea, that controlled for age and gender. Adults aged 26 years and older had lower odds of hydroxyurea use compared with the younger age categories (*p* = 0.0002). As reports of total barriers to hydroxyurea increased, the odds of hydroxyurea use decreased (*p* < 0.0001). The variable “not interested in (hydroxyurea)” from the providers’ perspective, was associated with lower odds of being on hydroxyurea. There was little variability in hydroxyurea use for the indication of recurrent pain, so that the odds associated with being on hydroxyurea were very high, with very wide confidence intervals. Gender was not associated with hydroxyurea use. Greater odds associated with empiric use [OR = 16.9, 95% CI (1.90–149.6), *p* = 0.011] and lower odds associated with diagnoses other than HgbSS and HgbSβ^0^ thalassemia [HgbSC OR = 0.291, 95% CI (0.097–0.874), other diagnoses OR = 0.069, 95% CI (0.006–0.737), *p* = 0.0285] approached significance.

Based on results from univariate models, diagnosis, HbF, total barriers and barriers contributing to unintentional nonadherence were entered into a multivariable model for provider prescription of hydroxyurea, that controlled for age and gender. Adults aged 26 years and older had lower odds of hydroxyurea use compared with the younger age categories (*p* = 0.0048). As reports of total barriers to hydroxyurea increased, the odds of hydroxyurea use decreased (*p* < 0.0001). Lower odds of prescribing hydroxyurea were associated with diagnoses other than HgbSS and HgbSβ^0^ thalassemia. Gender was not associated with hydroxyurea use.

An ad hoc analysis was performed to assess the potential contribution of site in the multivariable models. We performed multiple logistic regression analysis, where we used being on hydroxyurea as the binary outcome, with age, and site (binary variable—California sites (3) compared with other states (6 sites) as independent variables. Consistent with our main finding in Table 5, patients aged 26 years and older had significantly lower odds of being on hydroxyurea compared with the patients aged 12 years and younger. The results (available from the first author upon request) also showed that the patients from other states had significantly higher odds of being on hydroxyurea compared with the patients in California.

**TABLE 5:** Significant multivariable relations between patient/caregiver reports of being on hydroxyurea and provider prescription of hydroxyurea.

Model	Variable	Odds ratio (95%confidence interval)
Patient/Caregiver report of being on hydroxyurea		
	Age	
	12 years and younger	Reference
	13–17 years	1.9 (0.66–5.2)
	18–25 years	0.48 (0.15–1.53)
	26 years and older	0.13 (0.04–0.38)**
	Total Barriers	
	None	Reference
	1 barrier	0.08 (0.03–0.20)***
	2 or more barriers	0.19 (0.07–0.57)*
	Indication—Recurrent Pain	811.5 (87.7–7504.3)***
	Not Interested in hydroxyurea	0.06 (0.01–0.31)**
Provider prescription of hydroxyurea		
	Age	
	12 years and younger	Reference
	13–17 years	1.1 (0.56–2.1)
	18–25 years	0.65 (0.33–1.30)
	26 years and older	0.45 (0.25–0.78)*
	Total Barriers	
	None	Reference
	1 barrier	0.15 (0.09–0.26)***
	2 or more barriers	0.27 (0.14–0.52)**
	Diagnosis	
	HgbSS and HgbSβ^0^ thalassemia	Reference
	HgbSC	0.21 (0.10–0.41)***
	Other diagnoses	0.35 (0.13–0.90)

*p < 0.01, **p < 0.001, ***p < 0.0001.

## Discussion

To our knowledge, this is the largest real-world study to date that assesses hydroxyurea use for children and adults with SCD, from the perspectives of providers, adults, and caregivers of children. We found that hydroxyurea use was reported in over half of eligible patients from a large geographic region in the U.S., representing a range of sickle cell specialty clinical settings and practices. Provider and patient/caregiver reports about hydroxyurea use were consistent with one another, as was the importance of age as a factor associated with hydroxyurea use. Adults were significantly less likely than children, adolescents, and young adults to be on hydroxyurea, consistent with other studies (Sinha et al., 2018). Barriers to hydroxyurea were also factors associated with its use for both providers and patients/caregivers, with the likelihood of being on hydroxyurea decreasing with one or more barriers.

From the perspectives of adults with SCD and caregivers, the indication of recurrent pain was associated with higher odds of hydroxyurea use. Patient/caregiver perspectives that they were “not interested” in taking the medication were significantly associated with lower odds of its use. However, patients/caregivers also cited worry about side effects, not knowing enough about the medicine and providers not recommending hydroxyurea as barriers. Our results suggest that providers may need a greater appreciation of these important perspectives of adults and caregivers of children with SCD, in order to increase its use among eligible patients (Adeyemo et al., 2015; Okocha et al., 2022).

We adopted the intentional and unintentional medication nonadherence framework in the present study to enhance understanding of unique barriers to hydroxyurea for the SCD population (Hodges et al., 2020). We demonstrated that, even for patients on hydroxyurea, challenges to taking the medicine at the right time and forgetting were crucial unintentional barriers to adherence, consistent with other reports (Badawy et al., 2017; Jose et al., 2019; Hodges et al., 2020). Forgetfulness may be associated with neurocognitive deficits, including memory impairment and diminished executive functioning, that may occur in SCD (Feliu et al., 2011; Prussien et al., 2019; Martin et al., 2020). Healthcare providers should consider strategies that lessen the reliance on memory and planning to support adherence, including mobile apps (Creary et al., 2014; Badawy et al., 2016; Inoue et al., 2016; Makubi et al., 2016; Leonard et al., 2017; Curtis et al., 2019) and automated text message reminders (Estepp et al., 2014; Pernell et al., 2017) in addition to encouraging patients to incorporate hydroxyurea into their daily routines. Community health workers have also been utilized to facilitate adherence to treatment regimens, including hydroxyurea (Green et al., 2017).

Intentional barriers such as worry about side effects (Haywood et al., 2011; Oyeku et al., 2013; Hodges et al., 2020) and “tried and it did not work” were particularly important barriers for young adults and adults and possibly reflect a general lack of knowledge about hydroxyurea. Smith and colleagues (Smith et al., 2019) found a 158.8% increase in the number of patients initiating hydroxyurea after concerted education, suggesting the efficacy of educational interventions. Sinha and colleagues (Sinha et al., 2018) found that for 95 adults with SCD, those who could explain the mechanism of action for hydroxyurea and its benefits were more likely to be taking hydroxyurea and reported a more positive experience. Patient/family education to increase knowledge about hydroxyurea may thus mitigate barriers lending to intentional nonadherence (Pecker et al., 2018; Crosby et al., 2019). Our findings can assist providers in understanding choices and decisions of their families affected by SCD; guide individualized clinical discussions regarding hydroxyurea therapy; and help with developing tailored interventions to address barriers (Hodges et al., 2020). Shared decision making is an opportunity for the provider and patient/family to meet and discuss the purpose, benefits, mechanism of action, and side effects of hydroxyurea, as well as address any questions or concerns. Shared decision making may also enhance understanding and confidence in explaining and prescribing hydroxyurea on the part of the provider.

For providers, diagnoses other than sickle cell anemia (HgbSS or HgbS-β^0^ thalassemia), particularly HgbSC, were associated with lower odds of prescribing. Provider behavior was thus consistent with the NHLBI evidence-based guidelines in this regard (National Heart, Lung and Blood Institute, National Institutes of Health, 2014). However, adults 26 years and older had the most frequent reports of four or more visits to the ED for pain and interference from pain episodes experienced at home in the past year while being the least likely age group to receive prescriptions from their providers. Adults also were not prescribed hydroxyurea for empiric use, consistent with other studies (Thornburg et al., 2012). The NHLBI guidelines explicitly state that all patients with sickle cell anemia and their families should be educated about hydroxyurea and that infants, children and adolescents 9 months of age and older with sickle cell anemia should be offered hydroxyurea, regardless of clinical severity. The guidelines list a number of specific complications in adults with sickle cell anemia that should prompt treatment with hydroxyurea, perhaps creating confusion on the part of some providers about prescribing hydroxyurea empirically for adults. Implementing standard practices for hydroxyurea therapy across patients’ lifespans, including its empiric use, and considering their eligibility even if they do not have a diagnosis of sickle cell anemia, should be deliberated upon with future guidelines. Studies with larger sample sizes are needed to evaluate whether there are differences by age when hydroxyurea is prescribed for empiric use or prophylaxis rather than for disease related complications. Additional guidance about strategies for education about hydroxyurea, that emphasize avoidance of cumulative damage and the suffering associated with severe and frequent pain, may be helpful for providers, patients and families alike.

Cabana et al. (2019) reported that provider hesitancy in prescribing hydroxyurea, may be due to provider concerns about patient adherence to the medication, and this was the case for a handful of patients in our study. Furthermore, SCD providers in a previous study also expressed a lack of self-efficacy, particularly in identifying which patients may benefit from hydroxyurea, prescribing the correct dose, recognizing side effects, and discussing risks of the medication with patients/families (Cabana et al., 2019). Strengthening provider understanding and confidence in implementing the NHLBI SCD guidelines should not be taken for granted, even for providers with more experience with and support for SCD care, as in the PSCRC. The need to attend to the multiple factors that undermine quality of life and quality of care for adults with SCD remains more critical than ever (Kanter et al., 2020).

Short-term self-reported adherence with hydroxyurea was very high for those for whom it was prescribed in the present study and available laboratory values were consistent with patient/caregiver reports. Multiple measures of adherence that are triangulated are generally recommended, to optimally support shared decision-making (Lam and Fresco, 2015). However, in our study 25% of laboratory values were not recorded, perhaps given that we asked providers to report the values. This additional step may have been too burdensome in busy clinical settings. While we were pleasantly surprised at the alignment of patient/caregiver/provider assessments of adherence and of laboratory values that we had access to, future studies in real world settings must carefully weigh trade-offs in clinical relevance compared with feasibility of data capture.

Of note, over 40% of our patients/families were officially below the poverty line in the U.S., with low incomes and high household densities. Although not otherwise directly assessed in this study, we acknowledge that there are multiple social determinants of health that can impact uptake of and adherence with hydroxyurea and other aspects of treatment regimens (Power-Hays et al., 2020). Assessment of social determinants of health such as food and housing insecurity; employment and education; and experiences of racism and discrimination; should be routine, using valid and reliable measures. When assessments reveal negative impacts of social determinants of health, individuals with SCD and their families must be provided access to comprehensive team support and evidence-based therapies.

### Limitations

Several factors limit the generalizability of the present findings. First, despite the relatively large sample size, patients were from sites only in the western U.S. Individuals with SCD followed in other parts of the U.S. and across the globe might experience different barriers which may influence which interventions will be most helpful to increase hydroxyurea prescription and minimize barriers. Second, patients in this cohort were associated with clinics with an interest in improving SCD care as these centers were part of the PSCRC, that might not be the case in all clinics where patients with SCD are followed. Third, differences in access to comprehensive healthcare services may affect frequencies of ED visits and hospitalizations for pain.

In the present study, healthcare utilization was not associated with hydroxyurea use, unlike prior studies (Charache et al., 1995; Thornburg et al., 2012) that demonstrated that hydroxyurea use was associated with decreased frequency of pain and ED and inpatient admissions. This could be due to differences in study design and purpose. In addition, our data are limited by indication bias, as participants were offered hydroxyurea primarily for sickle cell related complications rather than for empiric use. Previous studies were randomized trials looking at the effects of hydroxyurea with a set medication start date. Our goal was to leverage a large regional collaborative to understand barriers to hydroxyurea use in real world clinical settings and establish an infrastructure for future quality improvement work on identifying the barriers to hydroxyurea use, regardless of length of time on hydroxyurea and dosage. Given how wide-ranging our sample sizes were across sites, we were not able to fully explore the influence of site. Such an analysis has a high potential for confounding and loss of statistical power and is beyond the scope of the present study. An ad hoc analysis did confirm that our findings held, that the patients aged 26 years and older have lower odds of being on hydroxyurea compared with younger patients even after adjusting for site differences. Our real-world evidence complements clinical trials with increased variability within our sample and by permitting a fuller understanding of patient/family and provider perspectives about hydroxyurea. Provider reasons for not prescribing hydroxyurea for “empiric use” could not be explored further given the term “empiric use” was not clearly defined. Lastly, these data were collected prior to the availability of other new therapies for SCD so we are unable to assess barriers to use of these newer therapies that might be different than barriers to hydroxyurea therapy.

## Conclusion

The purpose of this study was to describe factors associated with hydroxyurea use from the perspectives of providers, individuals with SCD, and families. We described clinical characteristics, healthcare utilization, barriers to hydroxyurea use and adherence from the perspectives of pediatric and adult patients with SCD and prescribing patterns and laboratory values in relation to hydroxyurea for eligible patients as reported by providers. Our findings support the importance of enhancing the knowledge base about hydroxyurea, particularly among adults living with SCD, as well as their providers. With the advent of new disease modifying therapies that will likely need to be utilized together, it will be important to spend adequate time in shared decision making so that individuals with SCD can optimally benefit from these therapies, including hydroxyurea. Addressing the barriers to hydroxyurea use among individuals with SCD can lead to steps to minimize similar barriers in the use of emerging and combination therapies for SCD. Sorting through issues with treatment guidelines and gaps in knowledge and shared decision making will support more effective implementation of disease modifying therapies in high and low-resource settings across the globe (Power-Hays and Ware, 2020).

## Data Availability

The raw data supporting the conclusion of this article will be made available by the authors, without undue reservation.
